# Computational model of damage-induced growth in soft biological tissues considering the mechanobiology of healing

**DOI:** 10.1007/s10237-021-01445-5

**Published:** 2021-03-26

**Authors:** Meike Gierig, Peter Wriggers, Michele Marino

**Affiliations:** 1 Institute of Continuum Mechanics, Leibniz University Hannover, An der Universität 1, 30823 Garbsen, Germany; 2 Department of Civil Engineering and Computer Science, University of Rome Tor Vergata, Via del Politecnico 1, 00133 Rome, Italy

**Keywords:** Soft biological tissue mechanics, Mechanobiology of healing, Damage-induced growth, Homogenized constrained mixtures

## Abstract

Healing in soft biological tissues is a chain of events on different time and length scales. This work presents a computational framework to capture and couple important mechanical, chemical and biological aspects of healing. A molecular-level damage in collagen, i.e., the interstrand delamination, is addressed as source of plastic deformation in tissues. This mechanism initiates a biochemical response and starts the chain of healing. In particular, damage is considered to be the stimulus for the production of matrix metalloproteinases and growth factors which in turn, respectively, degrade and produce collagen. Due to collagen turnover, the volume of the tissue changes, which can result either in normal or pathological healing. To capture the mechanisms on continuum scale, the deformation gradient is multiplicatively decomposed in inelastic and elastic deformation gradients. A recently proposed elasto-plastic formulation is, through a biochemical model, coupled with a growth and remodeling description based on homogenized constrained mixtures. After the discussion of the biological species response to the damage stimulus, the framework is implemented in a mixed nonlinear finite element formulation and a biaxial tension and an indentation tests are conducted on a prestretched flat tissue sample. The results illustrate that the model is able to describe the evolutions of growth factors and matrix metalloproteinases following damage and the subsequent growth and remodeling in the respect of equilibrium. The interplay between mechanical and chemo-biological events occurring during healing is captured, proving that the framework is a suitable basis for more detailed simulations of damage-induced tissue response.

## Introduction

When a soft biological tissue is injured, e.g., after trauma or surgical procedures, repair mechanisms within the tissue occur to rebuild functionality and integrity of the tissue. The repair process, from damage stimuli to the formation of new tissue, is yet not fully understood. It is a complex, interactive process at different length and time scales and invokes interactions between mechanical stimuli, chemical signals and biological species (Velnar et al. 2009; Thackham et al. 2008). The understanding is essential to prevent abnormal healing which is of special interest in clinical practice. For instance, clinicians want to avoid abnormal scar formation during wound healing or to maintain the restored functionality of blood vessels after vascular surgeries.

Soft biological tissue is mainly comprised of cells (e.g., fibroblasts, epithelial cells and muscle cells) embedded in a non-cellular structure that is comprised of numerous molecules (e.g., collagen, elastin, structural glycoproteins and proteoglycans) (Hay 1981; Alberts et al. 2002). The latter is named the extracellular matrix (ECM). Mechanical stimuli are carried mostly by ECM components. The primarily load bearing constituents within the ECM are collagen and elastin (Burton 1954; Cocciolone et al. 2018). While elastin ensures elasticity of the tissue at low strains, collagen acts as a reinforcing constituent at high strains (Roach and Burton 1957; Shadwick 1999).

As a result of supra-physiological loading, soft biological tissue is injured or damaged. At macroscopic level, this can be characterized mechanically by the stress-softening effect. Softening is mainly caused by damage in collagen losing its integrity (Weisbecker et al. 2012), whereas softening due to elastin damage was shown to be negligible (Weisbecker et al. 2013). At fibril scale, multiple inelastic mechanisms in collagen could be identified (Buehler 2006), involving slip-pulse, interstrand delamination and molecular covalent bond rupture (Marino 2016). Interstrand delamination (ID) is an irreversible collagen triple helix unfolding and corresponds to the sliding of a single polypeptide strand of the triple helix with respect to the other two. ID seems to be one of the most critical mechanism occurring to collagen molecules during tissue damage. The use of collagen hybridizing peptides (CHP) in combination with fluorescence and imaging techniques provides an experimental measure of collagen damage. In Zitnay et al. (2017), Marino et al. (2019), Lin et al. (2020), the existence of a relationship between the evolution of CHP-binding and tissue softening was shown, proving the significance of ID on the biomechanics of injured biostructures.

Even under normal physiologic conditions, cells continuously sense the state of ECM and deposit, rearrange or remove matrix (Lu et al. 2011). In turn, ECM influences migration, proliferation or differentiation of cells (Hoffman et al. 2011). ECM, specifically collagen, is continuously turned over by the activities of enzymes, such as matrix metalloproteinases (MMP), which degrade ECM and release growth factors (GF) (Bonnans et al. 2014). GF in turn contribute to the accommodation of new ECM, specifically of collagen (Forrester et al. 1991). These mechanisms contribute to the maintenance of homeostatic conditions or, in case of injury, to the tissue repair. Detailed descriptions of the regulatory mechanisms in tissues are given, e.g., by Humphrey et al. (2014), addressing the homeostasis process, and by Mouw et al. (2014) for non-healthy tissues.

Computational models are a promising tool to better understand the behavior of healing tissues and to identify key players in the chain of events (Humphrey 2013). Although numerous different types of biological soft tissues exist, they have in common the interaction of mechanical, chemical and biological events across space and time scales. Thus, computational models addressing healing should consider and couple these physically different mechanisms. The need of this coupling has recently been pointed out by Loerakker and Ristori in Loerakker and Ristori (2019), highlighting the importance for the modeling of cardiovascular tissue. A three-dimensional continuum model for the wound healing process has been presented by Buganza Tepole and Kuhl (2016). Their framework combines mechanical deformation with inflammatory signals and cell behavior and is monolithically solved. The chemical signal is given as a synthesized quantity that initiates a change in fibroblast density. Fibroblasts and inflammatory signals in turn alter the content of collagen. From the mechanical perspective, the key players in their framework are collagen and a non-collagenous matrix, together forming the ECM. Another model on continuum scale has been published by Escuer et al. (2019). The authors address the problem of restenosis, which is the pathological renarrowing of arteries after angioplasty. In their two-dimensional framework, the chain of events is initiated as soon as a stress value is exceeded. They consider the interaction of GF and MMP with vascular smooth muscle cells (VSMC), ECM and endothelial cells (EC). Beside continuum models, several agent-based models (ABM) exist to simulate healing. In contrast to continuum models and based on a set of rules, ABMs cannot capture the mechanical behavior but migration, proliferation and apoptosis of individual cells. They are often applied in the context of restenosis. Zun et al. (2017) published an ABM for a three dimensional, patient-specific, stented artery. To overcome the lack of mechanical information, ABMs have been coupled to finite element models, e.g., by Keshavarzian et al. (2018), Nolan and Lally (2018) and Li et al. (2019). The ABMs mostly consider EC and VSMC as cell populations and formulate sets of rules which represent chemical signals and the evolution of mediators such as MMPs. All the models mentioned above have in common that damage is treated at maximum as stiffness weakening. Inelastic deformations are not considered, although these would alter in turn G&R. In addition, these approaches generally consider homogeneous kinematic descriptions of growth, despite adopting more refined growth models of constrained mixtures which would account for the heterogeneous nature of soft tissues. A remarkable advancement in this direction is the homogeneous constrained mixture model proposed by Cyron et al. in Cyron et al. (2016), which gathers ease of implementation with the refinement of the description.

In this paper, a framework is proposed that considers the chain of chemo-bio-mechanical events involved in healing of soft tissues. Within the ECM, collagen is considered as the main structure becoming damaged. In contrast to the previously mentioned models, the recently available experimental evidence that collagen damage is in fact related to plastic deformations associated with interstrand delamination is incorporated and coupled with alterations in the mechanics of injured tissues. This mechanism is elected and used as damage stimulus. The repair process is modeled as initiated by the amount of collagen damage. As a consequence of the injury signal, MMP and GF species are synthesized, which is modeled by means of ordinary differential equations. These species contribute to the degradation (MMP) and deposition (GF) of collagen, leading in turn to tissue growth. At the continuum scale, the concentration of MMPs and GFs is coupled with a homogenized constrained mixture model, motivated by Cyron et al. (2016), to describe the change in tissue volume as a gross effect caused by mechanisms on molecular and cellular scale. With respect to a number of existing approaches (Buganza Tepole and Kuhl 2016; Escuer et al. 2019; Li et al. 2019; Nolan and Lally 2018), the proposed framework includes a consistent continuum-based biomechanical description and coupling of damage evolution, biological events and tissue growth and remodeling. Damage is not simply considered as an internal variable independent from mechanics, but it is initiated by overloads and determines tissue softening and permanent deformations. In turn, the kinematics of damage-induced tissue growth and remodeling is introduced consistently with the pre-existing elasto-plastic tissue state, determining the evolving configuration of healing biostructures in the respect of mechanical equilibrium.

## Methods

Soft tissue domain is modeled as continuous body occupying a region $\varOmega _0 \subset \mathbb {R}_3$ in reference configuration. The body moves in space and occupies at time *t* a region $\varOmega (t) \subset \mathbb {R}_3$, called current configuration. The deformation gradient is defined as a function of the displacement $\mathbf {u}$ as ${\mathbf{F}}= {\mathrm{Grad}}\left( {\mathbf{u}}\right) + {\mathbf{I}}$. It transforms a line element ${\mathrm {d}}{\mathbf {X}}\in \varOmega _0$ to a line element ${\mathrm {d}}{\mathbf {x}}\in \varOmega (t)$ via

$$\begin{aligned} \text{d}\mathbf {x}=\mathbf {F}\text{d}\mathbf {X}\, . \end{aligned}$$

In this paper, a non-collageneous matrix (superscript $i=\mathrm {m}$) and collagen fibers (superscript $i=\mathrm {c}$) are considered. A non-collageneous matrix is assumed to behave isotropically (Weisbecker et al. 2013). Collagen fibers are embedded in the matrix and induce anisotropy. These two constituents play a key role in the response of the tissue to mechanical stress (or stretch) (Burton 1954; Cocciolone et al. 2018). For the sake of simplicity, only these constituents are considered. However, the herein presented framework can readily be extended to further constituents.

### Multiplicative split of the deformation gradient

**Fig. 1:**
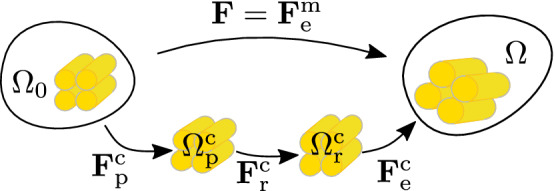
Configurations and multiplicative split of the deformation gradient

To account for damage, growth, remodeling and elastic mechanisms, the deformation gradient is multiplicatively decomposed

$$\begin{aligned} \mathbf {F}= & {} \mathbf {F}_\mathrm {e}^\mathrm {c}\, \mathbf {F}_\mathrm {r}^\mathrm {c}\, \mathbf {F}_\mathrm {p}^\mathrm {c}\\= & {} \mathbf {F}_\mathrm {e}^\mathrm {m}\, . \end{aligned}$$

The constituents experience the same total deformation but individual elastic and inelastic deformations, see Fig. 1. It is assumed that the matrix deforms purely elastic up to high strains since it is mainly comprised of elastin and has a high content of water and no split of the deformation gradient ${\mathbf {F}}_\mathrm {e}^\mathrm {m}$ is necessary. Only collagen is damaged due to unphysiological loading. The damage mechanism modeled is interstrand delamination what can be regarded as a source of plastic-like deformations in collagen due to the irreversible sliding between polypeptide strands. Thus, it is captured by a plastic collagen deformation gradient ${\mathbf {F}}_\mathrm {p}^\mathrm {c}$. Note that the damage formulation used herein differs from traditional damage mechanics (Kachanov 1986), where an effective strain energy function is reduced by multiplication of a scalar damage variable. The elastic deformation gradient captures at the one hand the elastic, reversible response of the tissue constituents due to mechanical loading and ensures mechanical equilibrium in the current configuration. Secondly, it compensates geometrically incompatible states in the intermediate configurations such that there are no gaps or overlaps between differential volume elements. In addition, the elastic deformation gradient contains the growth information, which is regarded as an elastic swelling under the constraint of constant spatial density, see Sec. 2.7. Remodeling of collagen is considered by introducing a remodeling deformation gradient ${\mathbf {F}}_{\mathrm {r}}^{\mathrm {c}}$, see Sec. 2.4. The right Cauchy Green tensor ${\mathbf {C}}= {\mathbf {F}}^{\mathrm {T}}{\mathbf {F}}$ can be defined for the elastic part as

$$\begin{aligned} \mathbf {C}_\mathrm {e}^i = \left( \mathbf {F}_\mathrm {e}^i \right) ^\mathrm {T}\mathbf {F}_\mathrm {e}^i \, , \end{aligned}$$

where $i=\mathrm {c},\mathrm {m}$. The direction of collagen fibers is $\mathbf {a}_0$ in the reference configuration with $\left\| \mathbf {a}_0 \right\| = 1$. The fiber directions in the intermediate and current configurations are obtained by mapping $\mathbf {a}_0$ with the corresponding deformation gradient. In addition, the direction vectors are normalized such that not only the deformation gradient can be multiplicative decomposed but also the stretch. Hence the direction vectors in the first intermediate $\mathbf {a}_\mathrm {p}$ and in the second intermediate configuration $\mathbf {a}_\mathrm {r}$ result as

$$\begin{aligned} \mathbf {a}_\mathrm {p}=\frac{\mathbf {F}_\mathrm {p}^\mathrm {c}\mathbf {a}_0}{\left\| \mathbf {F}_\mathrm {p}^\mathrm {c}\mathbf {a}_0 \right\| } \, , \qquad \mathbf {a}_\mathrm {r}=\frac{\mathbf {F}_\mathrm {r}^\mathrm {c}\mathbf {a}_\mathrm {p}}{\left\| \mathbf {F}_\mathrm {r}^\mathrm {c}\mathbf {a}_\mathrm {p}\right\| } \, . \end{aligned}$$

Accordingly, the stretches in fiber direction can be written as

$$\begin{aligned} \lambda _\mathrm {p}^\mathrm {c}= & {} \left\| \mathbf {F}_\mathrm {p}^\mathrm {c}\, \mathbf {a}_0 \right\| \, , \qquad \lambda _\mathrm {r}^\mathrm {c}= \left\| \mathbf {F}_\mathrm {r}^\mathrm {c}\, \mathbf {a}_\mathrm {p}\right\| \, ,\\ \lambda _\mathrm {e}^\mathrm {c}= & {} \left\| \mathbf {F}_\mathrm {e}^\mathrm {c}\, \mathbf {a}_\mathrm {r}\right\| \, , \qquad \lambda = \left\| \mathbf {F}\, \mathbf {a}_0 \right\| \, . \end{aligned}$$

For later use, structural tensors comprised of the dyadic product between the fiber orientation vectors are introduced:

$$\begin{aligned} \mathbf {M}_0 = \mathbf {a}_0 \otimes \mathbf {a}_0 \, , \qquad \mathbf {M}_\mathrm {p}= \mathbf {a}_\mathrm {p}\otimes \mathbf {a}_\mathrm {p}\, , \qquad \mathbf {M}_\mathrm {r}= \mathbf {a}_\mathrm {r}\otimes \mathbf {a}_\mathrm {r}\, . \end{aligned}$$

The isochoric first invariant of the matrix is introduced as

1 $$\begin{aligned} \bar{I}_1 = \mathrm {tr} \left( \bar{\mathbf {C}}_\mathrm {e}^\mathrm {m}\right) \, \text {, where} \qquad \bar{\mathbf {C}}_\mathrm {e}^\mathrm {m}= \det \left( \mathbf {C}_\mathrm {e}^\mathrm {m}\right) ^{-\frac{1}{3}} \mathbf {C}_\mathrm {e}^\mathrm {m}\, . \end{aligned}$$

Furthermore, the anisotropic invariant of $\mathbf {C}_\mathrm {e}^\mathrm {c}$ and $\mathbf {M}_\mathrm {r}$ is

2 $$\begin{aligned} I_\mathrm {e}^\mathrm {c}= \mathbf {C}_\mathrm {e}^\mathrm {c}: \mathbf {M}_\mathrm {r}\, , \end{aligned}$$

representing the squared elastic stretch of collagen fibers in the fiber direction.

### Balance of mass

The determinant of the deformation gradient $J=\det \mathbf {F}$ maps the volume element $\mathrm {d}V_0$ in the reference configuration of a body to a volume element $\mathrm {d}V$ in the current configuration via

3 $$\begin{aligned} \mathrm {d}V=J \, \mathrm {d}V_0 \, . \end{aligned}$$

The mass of a volume element is

4 $$\begin{aligned} \mathrm {d}m = \rho \, \mathrm {d}V = \rho _0 \, \mathrm {d}V_0 \, , \end{aligned}$$

where $\rho$ is the mass density in the current configuration, assumed to be (nearly) constant, $\rho \approx const$. This implies that, due to growth, the volume changes by exactly the same factor as the mass. In other words, in the spatial configuration the volume per unit mass remains constant, which is generally referred to as incompressible growth. In contrast, the mass density in the reference configuration $\rho _0$ is a function of time $\rho _0 = \rho _0(t)$. Thus, a change of the reference mass density directly translates into a change of current volume and mass. Inserting (3) into (4) yields

5 $$\begin{aligned} \rho = \frac{\rho _0}{J} \, . \end{aligned}$$

Note that the mass densities of the constituents are defined per total reference volume and not per volume of the constituents. Thus, the total reference mass density is the sum of the reference mass densities of the constituents:

6 $$\begin{aligned} \rho _0 = \rho _0^\mathrm {c}+ \rho _0^\mathrm {m}\, . \end{aligned}$$

Herein, the contributions of GF and MMP are neglected because the mass of these species is more than six orders of magnitude smaller.

The mass of region $\varOmega$ is obtained by integrating the infinitesimal mass over that region

$$\begin{aligned} m = \int _{\varOmega } \rho \mathrm {d}V \, . \end{aligned}$$

In the presented theory, tissue growth is caused by volumetric growth only. Based on the slow-growth assumption (Goriely 2017), mass fluxes are not considered. Moreover, new tissue is produced with the same spatial mass density as of the existent tissue, yielding

7 $$\begin{aligned} \dot{m} = \frac{\mathrm {d}}{\mathrm {d}t} \int _{\varOmega } \rho \mathrm {d}V = \int _{\varOmega } \rho \gamma \mathrm {d}V \, , \end{aligned}$$

where $\gamma$ is a growth rate function. Inserting (4) in (7), the rate of mass in the reference configuration is obtained:

8 $$\begin{aligned} \dot{m} = \frac{\mathrm {d}}{\mathrm {d}t} \int _{\varOmega _0} \rho _0 \mathrm {d}V_0 = \int _{\varOmega _0} \rho _0 \gamma \mathrm {d}V_0 \, . \end{aligned}$$

Localization finally yields the rate equation of the reference mass density

9 $$\begin{aligned} \dot{\rho }_0 = \rho _0 \gamma \, . \end{aligned}$$

Equation (5) implies that a change in the reference density translates into a change in volume and thus an appropriate growth rate function $\gamma$ has to be chosen to describe the volumetric growth.

### Helmholtz free energy and stresses

An invariant-based Helmholtz free energy function $\varPsi$ for the mixture describes the stress response of the tissue. The isotropic properties of the matrix are assumed to depend on the first invariant $\bar{I}_1$ in (1). Furthermore, the stress depends on the elastic stretch of collagen and the fiber direction via the anisotropic invariant $I_\mathrm {e}^\mathrm {c}$ in (2). The free energy of the mixture is additively decomposed:

10 $$\begin{aligned} \varPsi = \varPsi ^\mathrm {se} + \varPsi ^\mathrm {L} = \rho _0^\mathrm {c}W^\mathrm {c}+ \rho _0^\mathrm {m}W^\mathrm {m}+ \rho _0 W^\mathrm {L} \, . \end{aligned}$$

Here, a non-constrained strain energy term $\varPsi ^\mathrm {se}$ is introduced made up by the contributions from collagen and the matrix. The term $\varPsi ^\mathrm {L}$ accounts for the interactions between the individual mass increments in the constrained mixtures under a nearly incompressible elastic behavior of the mixture. $\varPsi ^\mathrm {L}$ constrains the volume change to be equal to the growth deformation, see the following Sec. 2.7. The strain energies $W^i$, $i=\mathrm {c,m,L}$ are defined per unit mass and multiplied by the reference mass densities per unit volume of the whole tissue. The strain energy $\varPsi$ and the components $\varPsi ^\mathrm {se}$ and $\varPsi ^\mathrm {L}$ are thus defined per unit reference volume.

The 2nd Piola-Kirchhoff stress (2nd PK) follows from

$$\begin{aligned} \mathbf {S}= 2 \frac{\partial \varPsi }{\partial \mathbf {C}} = 2 \sum _{i=\mathrm {c},\mathrm {m}} \rho _0^i \frac{\partial W^i}{\partial \mathbf {C}} + 2 \rho _0 \frac{\partial W^\mathrm {L}}{\partial \mathbf {C}} = \sum _{i=\mathrm {c},\mathrm {m}} \phi ^i \mathbf {S}^i + \mathbf {S}^\mathrm {L} \, . \end{aligned}$$

The mass fractions (constituent mass per total mass) are introduced as $\phi ^i=m^i/m$. The contribution to the stress due to volumetric growth $\mathbf {S}^\mathrm {L}$ is described for the whole mixture. The $\mathbf {S}^i$ can be interpreted as the $2^\mathrm {nd}$ PK stresses of the constituents if the whole volume was filled by a single *i*th constituent only.

$$\begin{aligned} \mathbf {S}^i = 2\frac{\rho _0^i}{\phi ^i} \frac{\partial W^i}{\partial \mathbf {C}} \qquad \text {and} \qquad \mathbf {S}^\mathrm {L} = 2\rho _0 \frac{\partial W^\mathrm {L}}{\partial \mathbf {C}} \, . \end{aligned}$$

The Cauchy stress can be obtained via a push forward of the $2^\mathrm {nd}$ PK as

$$\begin{aligned} \varvec{\sigma }= \frac{1}{J} \mathbf {F}\mathbf {S}\mathbf {F}^\mathrm {T}= \sum _{i=\mathrm {c},\mathrm {m}} \phi ^i \varvec{\sigma }^i + \varvec{\sigma }^\mathrm {L} \, . \end{aligned}$$

with

$$\begin{aligned} \varvec{\sigma }^i = \frac{1}{J} \mathbf {F}\mathbf {S}^i \mathbf {F}^\mathrm {T}\qquad \text {and} \qquad \varvec{\sigma }^\mathrm {L} = \frac{1}{J} \mathbf {F}\mathbf {S}^\mathrm {L} \mathbf {F}^\mathrm {T}\, . \end{aligned}$$

To obtain the contribution of the *i*th constituent to the Cauchy stress, the $\varvec{\sigma }^i$ need to be weighted by the mass fraction $\phi ^i$ of the *i*th constituent.

### Remodeling formulation

Besides the addition of mass through growth, collagen is continuously degraded and deposited to maintain or return to a stable mechanical state. New collagen is thereby deposited in the ECM at a certain stretch, which is called pre-stretch $\lambda _\mathrm {pre}^\mathrm {c}$. The stretch difference between existing collagen and newly formed collagen drives mass turnover. To capture this mass turnover, an isochoric remodeling deformation gradient is introduced

$$\begin{aligned} \mathbf {F}_\mathrm {r}^\mathrm {c}= \lambda _\mathrm {r}^\mathrm {c}\mathbf {M}_\mathrm {p}+ \frac{1}{\sqrt{\lambda _\mathrm {r}^\mathrm {c}}} \left( \mathbf {I}- \mathbf {M}_\mathrm {p}\right) \, . \end{aligned}$$

The rate of the remodeling stretch in fiber direction $\dot{\lambda }_\mathrm {r}^\mathrm {c}$ is, motivated by the remodeling approach in Grytsan et al. (2017), governed by the difference between the existing elastic collagen stretch and the pre-stretch

11 $$\begin{aligned} \dot{\lambda }_\mathrm {r}^\mathrm {c}= k_\mathrm {r}\frac{\lambda _\mathrm {e}^\mathrm {c}-\lambda _\mathrm {pre}^\mathrm {c}}{\lambda _\mathrm {pre}^\mathrm {c}-1} \, , \end{aligned}$$

where $k_\mathrm {r}$ is a parameter.

### Damage formulation

Damage is described in the framework of continuum damage mechanics. At the macroscale, stress softening is modeled. Tissue mechanics is governed by a set of internal variables which is descriptive for the average damage in collagen fibers within the microstructure of the tissue. The formulation is based on the damage model proposed in Marino et al. (2019). However, for consistency with the proposed growth framework, the approach is slightly adapted by using the plastic stretch rather than the square of the plastic stretch as internal variable and by assuming the plastic deformation gradient to be incompressible.

The maximum collagen stretch $\lambda ^\mathrm {c}_{\max }(t)$ contributing to damage is the maximum of the tissue stretch in the direction of the collagen fibers $\lambda (\tau )$ within the time range $\left[ 0,t \right]$ divided by the remodeling stretch $\lambda _\mathrm {r}(t)$

$$\begin{aligned} \lambda ^\mathrm {c}_{\max }(t) = \frac{\max _{\tau \in \left[ 0,t\right] } \lambda (\tau )}{\lambda _\mathrm {r}^\mathrm {c}(t)}\, . \end{aligned}$$

The remodeling stretch $\lambda _\mathrm {r}^\mathrm {c}$ does not contribute to damage and is removed from the total stretch $\lambda$.

Once the maximum stretch exceeds a physiological threshold $\bar{\lambda }_\mathrm {p}$, damage evolves. The damage variable $d \in [0,1]$ is zero as long as no damage occurs and reaches its maximum $d=1$ when the collagen is completely damaged. Above a stretch threshold $\bar{\lambda }_\mathrm {p}$ and as long as $d \in [0,1]$, damage is proportional to the difference between maximum collagen stretch and the stretch threshold

12 $$\begin{aligned} d = {\left\{ \begin{array}{ll} m_\mathrm {p}\left( \lambda _{\max }^\mathrm {c}- \bar{\lambda }_\mathrm {p}\right) &{} \mathrm {if} \, \lambda ^\mathrm {c}_{\max } > \bar{\lambda }_\mathrm {p}\, \wedge \, d < 1 \\ 0 &{} \mathrm {otherwise} \end{array}\right. } \, . \end{aligned}$$

Following the continuum damage theory, e.g., by Simo and Hughes (2006), damage is irreversible and thus $\dot{d} \ge 0$ must hold. This is, through the definition of $\lambda ^\mathrm {c}_{\max }$ and with $m_\mathrm {p}=const.$, automatically fulfilled and the damage rate reads

$$\begin{aligned} \dot{d} = {\left\{ \begin{array}{ll} m_\mathrm {p}\dot{\lambda }^\mathrm {c}_{\max } &{} \mathrm {if} \, \lambda ^\mathrm {c}_{\max } > \bar{\lambda }_\mathrm {p}\, \wedge \, d < 1 \\ 0 &{} \mathrm {otherwise} \end{array}\right. } \, . \end{aligned}$$

The flow rule is governed by the evolution of the maximum stretch, the damage variable *d* and the elastic collagen stretch $\lambda _\mathrm {e}^\mathrm {c}$ in fiber direction

13 $$\begin{aligned} \dot{\lambda }_\mathrm {p}^\mathrm {c}= {\left\{ \begin{array}{ll} d \frac{\dot{\lambda }^\mathrm {c}_{\max }}{\lambda _\mathrm {e}^\mathrm {c}} &{} \mathrm {if} \, \lambda _\mathrm {e}^\mathrm {c}> 1 \\ 0 &{} \mathrm {otherwise} \end{array}\right. }\, , \end{aligned}$$

yielding the rate of the plastic stretch in the direction of the collagen fiber. Increasing plastic stretch inherits a progressive decrease in the elastic stretch $\lambda _\mathrm {e}^\mathrm {c}$. The maximum amount of plastic stretch is limited by the case that the elastic stretch equals one, $\lambda _\mathrm {e}^\mathrm {c}=1$.

The plastic deformation is assumed to be anisotropic (aligned with collagen fibers direction) and incompressible such that an increase in volume is solely a consequence of healing. Consequently, the plastic deformation gradient is defined as

$$\begin{aligned} \mathbf {F}_\mathrm {p}^\mathrm {c}= \lambda _\mathrm {p}^\mathrm {c}\mathbf {M}_0 + \frac{1}{\sqrt{\lambda _\mathrm {p}^\mathrm {c}}} \left( \mathbf {I}- \mathbf {M}_0 \right) \, . \end{aligned}$$

The biological model, describing the reaction of the tissue to damage, is developed assuming that the problem can be divided into a damaging phase, lasting seconds to minutes, and a healing (growing) phase, lasting hours to years. The time $t^{*}$ denotes the time at which damage-related variables (*d* and $\lambda _\mathrm {p}^\mathrm {c}$) have reached a steady state. In other words, the present framework is conceived for injurious events, e.g., caused by trauma or surgical procedures. Once the tissue is damaged, for $t> t^{*}$, no further damage occurs and the healing phase begins. The translation of the damage stimulus into the alteration of the biochemical environment (i.e., MMPs and GFs concentrations) is introduced in the next section.

### Biological model

**Fig. 2:**
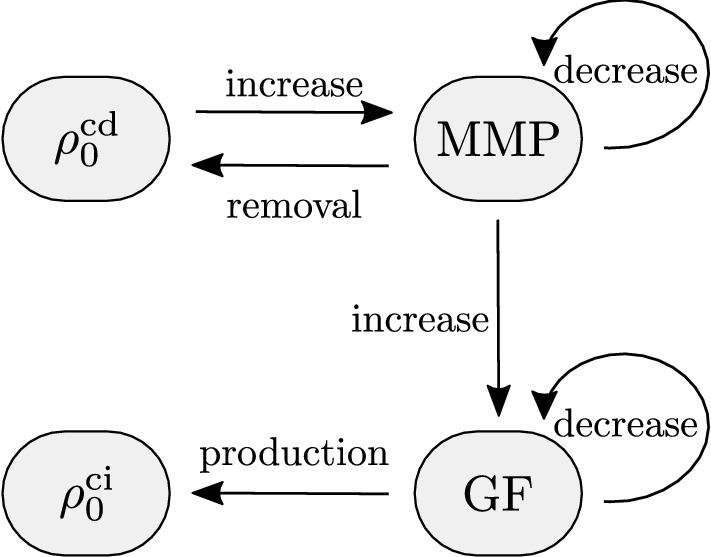
Schematic illustration of the role of MMP and GF for the collagen mass turnover initiated by the presence of damaged tissue

With equations (6) and (9), the growth rate function can be written as

$$\begin{aligned} \gamma = \frac{\dot{\rho }_0}{\rho _0} = \frac{1}{\rho _0} \left( \dot{\rho }_0^\mathrm {c}+ \dot{\rho }_0^\mathrm {m}\right) \, . \end{aligned}$$

The induced damage causes a stress softening at the macroscale and initiates mechanisms at the microscale which in turn cause addition of mass and thus growth at the macroscale (see the following Sec. 2.7). Thus, the amount of damage correlates with the amount of tissue that has to be repaired. Since only collagen damage and repair is considered, $\dot{\rho }_0^\mathrm {m}= 0$ and hence (9) simplifies to

$$\begin{aligned} \dot{\rho }_0 = \dot{\rho }_0^\mathrm {c}\, . \end{aligned}$$

Note that the change of the reference density is a “net” mass change. Under homeostatic conditions, the tissue is continuously turned over with $\dot{\rho }_0=0$ since mass production and removal balance each other. Injury disturbs this balance and hence $\dot{\rho }_0 \ne 0$ anymore.

To quantify damaged and intact collagen, the reference mass density of collagen is additively split into

$$\begin{aligned} \rho _0^\mathrm {c}= \rho _0^{\mathrm {c}\mathrm {i}}+ \rho _0^{\mathrm {c}\mathrm {d}}\, , \end{aligned}$$

where the superscripts (${\mathrm {c}\mathrm {i}}$) and (${\mathrm {c}\mathrm {d}}$) mean *collagen intact* and *collagen damaged*, respectively. The amount of collagen to be repaired at the beginning of the healing phase (at $\tau =t^{*}$) depends, on the one hand, on how damaged the collagen is and, on the other hand, on the amount of collagen itself. Thus, the initial values of damaged $\rho _0^{{\mathrm {c}\mathrm {d}}}|_{\tau =t^{*}} = \rho _0^{{\mathrm {c}\mathrm {d}}*}$ and intact $\rho _0^{{\mathrm {c}\mathrm {i}}}|_{\tau =t^{*}} = \rho _0^{{\mathrm {c}\mathrm {i}}*}$ collagen mass density are introduced as a function of the damage variable $d|_{\tau = t^{*}} = d^{*}$ obtained at the end of the damaging phase (see Sec. 2.5):

14 $$\begin{aligned} \rho _0^{{\mathrm {c}\mathrm {d}}*}= & {} d^{*} \rho _0^{\mathrm {c}*} \, , \end{aligned}$$

15 $$\begin{aligned} \rho _0^{{\mathrm {c}\mathrm {i}}*}= & {} (1-d^{*}) \rho _0^{\mathrm {c}*} \, . \end{aligned}$$

The collagen mass density is $\rho _0^{\mathrm {c}*}$ directly after the damage phase. Since the density does not change during the damaging phase, it corresponds to the initial collagen density. Note that the contribution of collagen to the strain energy per unit volume (10) is not further divided into a contribution for damaged and intact collagen, as has been done, e.g., by He et al. (2019) and Zuo et al. (2020).

The model introduced for describing the main biological cascade of events following damage is schematically depicted in Fig. 2. For MMP, the concentration variable *M* and for GF, variable *G* are introduced. Following damage of soft tissue, MMP concentration is upregulated and decreases with time back toward the homeostatic concentration $M_0$, see Bendeck et al. (1994). To reproduce these observations, a production term is defined as a function of damaged collagen and combined with a reduction term to describe a natural decay toward $M_0$

16 $$\begin{aligned} \dot{M} = m_1 \frac{ \rho _{0}^{\mathrm {c}\mathrm {d}}}{\rho _0^{\mathrm {c}*}} - m_2 \left( M-M_0 \right) \, . \end{aligned}$$

The presented approach introduces two parameters, $m_1$ quantifying the production of MMP due to the presence of damaged collagen and $m_2$ quantifying the natural reduction. Although at homeostatic conditions, concentration $M_0$ induces a continuous turnover of collagen, this is not explicitly modeled in the biological description, but its mechanical effect is taken into account within the remodeling inelastic deformation. Only the biological effect outside the homeostatic condition, that is collagen degradation and production for $M \ne M_0$, is considered.

The increased MMP concentration in turn attracts GF to the site of damage. Accordingly, the concentration of GF increases as well (Cromack et al. 1987), but delayed with respect to the MMP concentration. After reaching a peak, the GF concentration decreases toward the homeostatic level $G_0$. Consequently, the GF rate is defined with a production term, function of the accumulated MMP $I_M$, and a reduction term, function of GF concentration:

17 $$\begin{aligned} \dot{G} = g_1 I_M - g_2 \left( G-G_0 \right) \, . \end{aligned}$$

Parameter $g_1$ quantifies the production of GF from MMP, whereas $g_2$ describes the natural decay of GF. The function of accumulated MMP ($I_M$) is obtained by integrating the MMP concentration $M-M_0$ over the time range $\tau \in \left[ t^*,t\right]$ where $\tau =t^*$ is the time the healing phase begins, i.e., MMP concentration starts to increase:

18 $$\begin{aligned} I_M = I_M(t) = \left( \frac{1}{t-t^*} \underbrace{\int \limits _{t^*}^t M(\tau ) \mathrm {d}\tau }_{=:i_M} - M_0 \right) \exp \left( -\frac{t-t^*}{t_\mathrm {decay}} \right) \, . \end{aligned}$$

The exponential time-dependent function is introduced such that

$$\begin{aligned} \lim _{t \rightarrow \infty } I_M(t) = 0 \, . \end{aligned}$$

This term introduces a saturation behavior in the model representing the tendency of biological systems to be non-responsive to continuous stimuli in time. Here, the parameter $t_\mathrm {decay}$ governs, how fast $I_M$ tends toward zero.

Since MMP upregulation causes degradation of collagen, damaged collagen is degraded as a function of MMP:

19 $$\begin{aligned} \dot{\rho }_{0}^{\mathrm {c}\mathrm {d}}= {\left\{ \begin{array}{ll} k_\mathrm {d1} \left( \exp \left( - k_\mathrm {d2} \frac{M - M_0}{M_0} \right) -1 \right) &{} \mathrm {if} \, M > M_0 \\ 0 &{} \mathrm {otherwise} \end{array}\right. } \, . \end{aligned}$$

As shown by Van Doren (2015), MMP act on susceptible sites and not everywhere on fibrils. Therefore, since susceptible sites are likely more exposed in damaged collagen fibrils, only the effect of MMP on damaged collagen is modeled. The exponential function describes that less damaged tissue is removed faster than a higher amount of damaged tissue, see Fig. 4e. GF drives production of intact collagen at the side of damage. Thus, the deposition of intact collagen density is modeled as a function of GF

20 $$\begin{aligned} \dot{\rho }_{0}^{\mathrm {c}\mathrm {i}}= k_\mathrm {i1} \left( 1 - \exp \left( -k_\mathrm {i2} \frac{G-G_0}{G_0} \right) \right) \, . \end{aligned}$$

It is worth highlighting that initial conditions for (19) and (20), for integration over $\tau \in [t^{*}, t]$, are given in (14) and (15), respectively. Alterations in collagen densities translate in tissue volume changes, i.e., tissue growth, as introduced in the next section.

### Growth formulation

For a constant spatial density, growth describes the change of volume due to the change of mass. Growth depends on the MMP and GF activities on microscale that lead to deposition and degradation of ECM, see Sec. 2.6. Assumed herein is that the change of mass is solely due to the healing process. Via the balance of mass (7), mass change can be described by a change of the reference mass density as in (9). Due to the high amount of water in soft tissue, it is reasonable to consider soft tissue as nearly incompressible such that volume changes are only caused by growth. In line with this assumption, the property of a nearly constant density, $\rho (t)\approx \rho _0(0)$, is inserted into (5) yielding the constraint $\det (\mathbf {F}) \approx \frac{\rho _0}{\rho _0(0)}$. For $W^\mathrm {L}$ in the free energy (10), a constitutive equation is introduced for the volumetric deformation via a penalty-type approach, see Gierig et al. (2019):

21 $$\begin{aligned} W^\mathrm {L}(J) = \frac{\kappa }{2} \left( J - \frac{\rho _0}{\rho _0(0)} \right) ^2 \, , \end{aligned}$$

where $\kappa$ is a parameter. In the limit $\kappa \rightarrow \infty$, incompressibility is enforced.

Following Braeu et al. (2019), the deposition of new mass during growth can be interpreted as elastic swelling. In other words, since the elastic parts of the deformation gradient ($\mathbf {F}_\mathrm {e}^\mathrm {c}$ and $\mathbf {F}_\mathrm {e}^\mathrm {m}$) are directly affected by the deposition of mass, growth is associated with a change of the material strain energy. In this case, volume will automatically increase predominantly in the directions of lowest stiffness. As a matter of fact, the direction of growth is determined via minimization of the potential energy, favoring the more compliant directions. The concept is not explained herein in detail, it is rather referred to the original paper (Braeu et al. 2019). Briefly, looking at an isolated differential volume element of the mixture, the traction free configuration evolves when growth occurs. The traction free configuration is in the case of several constituents a configuration, in which the average stress of the constituents weighted with their respective mass fractions equals zero. The evolution of the traction free configuration is automatically enforced by the kinematic split in (1) and stationary conditions of a total potential energy with a free energy as in (10) and $\varPsi _\mathrm {L}$ as in (21), see the following Sec. 3.

## Numerical implementation

**Fig. 3:**
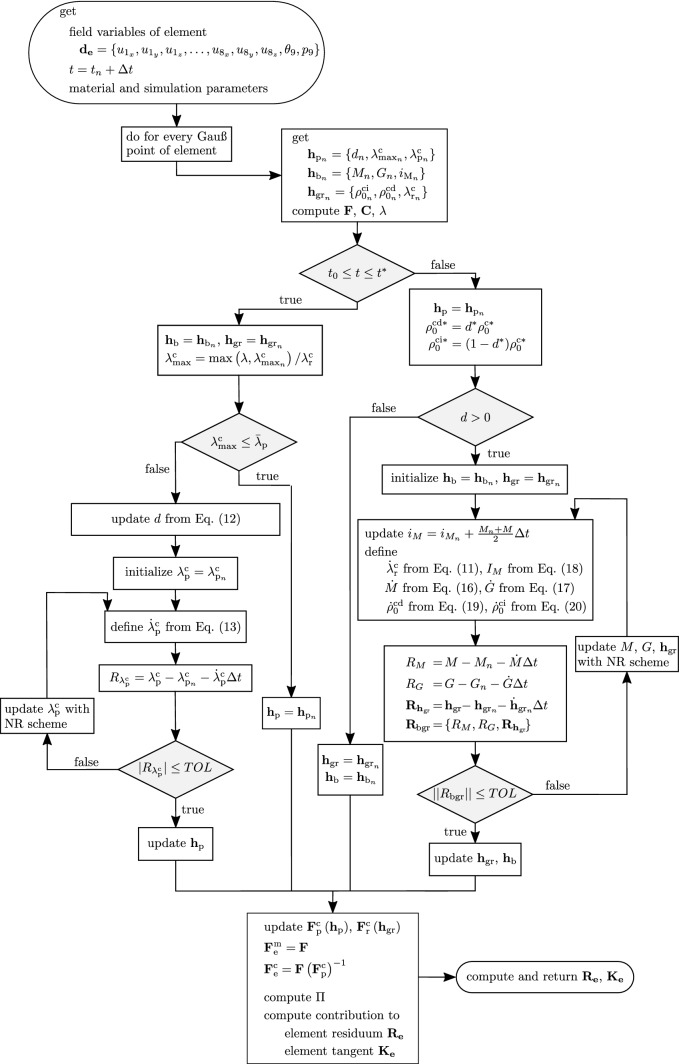
Flowchart of the routine at Gauß point level, abbreviation NR: Newton-Raphson

Numerical simulations on the basis of the theoretical model developed in the last sections are performed within the finite element method. The associated code is automatically generated using the Mathematica based tool AceGen (Korelc and Wriggers 2016). The output code is generated in C language for the finite element environment AceFEM.

To account for volume change in the growth process, the finite element is based on a Hu-Washizu variational formulation, with respect to the volumetric term:

22 $$\begin{aligned} \varPi \left( \mathbf {u}, p, \theta \right)= & {} \int _{\varOmega _0} \left[ \rho _0^\mathrm {c}W^\mathrm {c}\left( \mathbf {u}\right) + \rho _0^\mathrm {m}W^\mathrm {m}\left( \mathbf {u}\right) \right. \nonumber \\&\left. + \, \rho _0 \left( W^\mathrm {L}\left( \theta \right) + p \left( J - \theta \right) \right) \right] \mathrm {d}V \, . \end{aligned}$$

The potential energy is a three-field functional with independent variables displacement $\mathbf {u}$, pressure *p* and dilatation $\theta$. Variable *p* is a Lagrangian parameter which enforces the equivalence (in a weak sense) between $\theta$ and *J*. In turn, $\theta$ is controlled by variations of $\rho _0$ through $W^\mathrm {L}(\theta )$, see (21). The formulation is implemented in a finite element formulation for a nine-noded hexahedral mixed element. Each of the eight nodes at the edges has three degress of freedom presenting the components of the displacement vector $\mathbf {u}$. The node in the middle of the element has two degrees of freedom presenting $\theta$ and *p*. Accordingly, the element represents a H1-P0 element, i.e., trilinear shape functions for the displacement field and a constant ansatz for the pressure and volumetric strain. The internal variables of the model are split into three sets: $\mathbf {h}_\mathrm {p} = \{ d,\lambda ^\mathrm {c}_{\max },\lambda _\mathrm {p}^\mathrm {c} \}$ corresponding to the plastic behavior, $\mathbf {h}_\mathrm {b} = \{ M,G,i_\mathrm {M} \}$ for the biological species and $\mathbf {h}_\mathrm {g} = \{ \rho _0^\mathrm {ci},\rho _0^\mathrm {cd} , \lambda _\mathrm {r}^\mathrm {c}\}$ for the growth behavior. A time discretization based on a backward Euler scheme is introduced, with the internal and field variables at the previous time step $t_n$ denoted by the index *n*. Locally at the Gauß  points, implicit Newton-Raphson iterations are conducted to compute the internal variables.

Since damage and growth occur at different time scales, these mechanisms are modeled in a staggered and consecutive way, split by the time values $t_0$ and $t^{*}$. To consider tissue prestretch, an initial simulation is conducted to apply physiological loading conditions and let the tissue remodel such that at time $t_0$ it has reached a stable physiological state. No damage and evolution of biological species are considered in this prestretch simulation (i.e., for $t < t_0$). As previously mentioned, $t^{*}$ is a simulation parameter at which steady-state conditions for damage shall be reached. Therefore, it depends on loading conditions and other damage-related material constants. For $t_0 \le t \le t^*$, the internal variables for the molecular species and growth are held constant. Afterwards, for $t > t^*$, the damage variables are held constant and used for computing the initial conditions of the biological events inducing growth and remodeling, defining also the permanent tissue deformation determined by the damage event. A flowchart of the material routine at Gauß  point level is presented in Fig. 3.

Once the local material routine has reached convergence, the global problem, finding the field variables, is as well solved using an implicit Newton-Raphson algorithm at every time step. The local and global Newton-Raphson routines are solved in a nested way until both converge to a prescribed tolerance.

## Results

**Table 1:** Parameters for the biological species study

parameter	value	unit	parameter	value	unit	parameter	value	unit
$M_0$	$5.6 \times 10^{-5}$	$\frac{{\rm kg}}{{\rm m}^3}$	$g_1$	$10^{-4}$	$\frac{1}{{\rm s}}$	$k_\mathrm {d2}$	10.0	–
$G_0$	$3.5 \times 10^{-5}$	$\frac{{\rm kg}}{{\rm m}^3}$	$g_2$	$4.63 \times 10^{-5}$	$\frac{1}{{\rm s}}$	$k_\mathrm {i1}$	$10^{-4}$	$\frac{{\rm kg}}{{\rm m}^3\,{\rm s}}$
$m_1$	$4 \times 10^{-11}$	$\frac{{\rm kg}}{{\rm m}^3}$	$t_\mathrm {decay}$	1/2 year	s	$k_\mathrm {i2}$	2.0	–
$m_2$	$10^{-6}$	$\frac{1}{{\rm s}}$	$k_\mathrm {d1}$	$10^{-4}$	$\frac{{\rm kg}}{{\rm m}^3\,{\rm s}}$

**Table 2:** Parameters for the uniaxial tension test

parameter	value	unit	parameter	value	unit	parameter	value	unit
$l_x$	6	$\mathrm {mm}$	$\rho _0$	1050.0	$\frac{{\rm kg}}{{\rm m}^3}$	$m_2$	$10^{-6}$	1/s
$l_y$	3	$\mathrm {mm}$	$m_\mathrm {p}$	2.5	–	$g_1$	$10^{-4}$	1/s
$l_z$	0.225	$\mathrm {mm}$	$\bar{\lambda }_\mathrm {p}$	1.3	–	$g_2$	$4.63 \times 10^{-5}$	1/s
$\mu$	115.0	$\frac{\rm J}{\rm kg}$	${\lambda }^\mathrm {c}_\mathrm {pre}$	1.062	–	$t_\mathrm {decay}$	1/2 year	s
$k_1$	2.0	$\frac{\rm J}{\rm kg}$	$k_\mathrm {rem}$	0.8	1/year	$k_\mathrm {d1}$	$10^{-4}$	$\frac{{\rm kg}}{{\rm m}^3\,{\rm s}}$
$k_2$	3.2	–	$M_0$	$5.6 \times 10^{-5}$	$\frac{{\rm kg}}{{\rm m}^3}$	$k_\mathrm {d2}$	10.0	–
$\kappa$	$10^7$	$\frac{\rm J}{\rm kg}$	$G_0$	$3.5 \times 10^{-5}$	$\frac{{\rm kg}}{{\rm m}^3}$	$k_\mathrm {i1}$	$2.0 \times 10^{-4}$	$\frac{{\rm kg}}{{\rm m}^3\,{\rm s}}$
$\phi _0^\mathrm {c}$	0.8	–	$m_1$	$4 \times 10^{-11}$	$\frac{{\rm kg}}{{\rm m}^3\,{\rm s}}$	$k_\mathrm {i2}$	2.0	–

**Table 3:** Parameters for the indentation test

parameter	value	unit	parameter	value	unit	parameter	value	unit
$l_x$	6	$\mathrm {cm}$	$\rho _0$	1050.0	$\frac{{\rm kg}}{{\rm m}^3\,{\rm s}}$	$m_2$	$10^{-6}$	1/s
$l_y$	6	$\mathrm {cm}$	$m_\mathrm {p}$	4.5	–	$g_1$	$2 \times 10^{-4}$	${\frac{1}{\rm s}}$
$l_z$	1.2	$\mathrm {cm}$	$\bar{\lambda }_\mathrm {p}$	1.1	–	$g_2$	$6.94 \times 10^{-5}$	${\frac{1}{\rm s}}$
$\mu$	115.0	${\frac{\rm J}{\rm kg}}$	${\lambda }^\mathrm {c}_\mathrm {pre}$	1.062	–	$t_\mathrm {decay}$	1/2 year	$\mathrm {s}$
$k_1$	10.0	${\frac{\rm J}{\rm kg}}$	$k_\mathrm {rem}$	0.8	1/year	$k_\mathrm {d1}$	$10^{-4}$	${\frac{\rm kg}{\rm m^3 \rm s}}$
$k_2$	3.2	–	$M_0$	$5.6 \times 10^{-5}$	${\frac{\rm kg}{\rm m^3}}$	$k_\mathrm {d2}$	10.0	–
$\kappa$	$10^8$	${\frac{\rm J}{\rm kg}}$	$G_0$	$3.5 \times 10^{-5}$	${\frac{\rm kg}{\rm m^3}}$	$k_\mathrm {i1}$	$10^{-4}$	${\frac{\rm kg}{\rm m^3 \rm s}}$
$\phi _0^\mathrm {c}$	0.8	–	$m_1$	$4 \times 10^{-11}$	${\frac{\rm kg}{\rm m^3 \rm s}}$	$k_\mathrm {i2}$	2.0	–

The strain energy for collagen is chosen as an exponential function depending on the anisotropic invariant $I_\mathrm {e}^\mathrm {c}$, cf. (2)

23 $$\begin{aligned} W^\mathrm {c}= \frac{k_1}{2k_2} \left( \mathrm {e}^{k_2 \langle I_\mathrm {e}^\mathrm {c}-1 \rangle ^2}-1\right) \, , \end{aligned}$$

where $\langle \cdot \rangle$ denotes the Macaulay bracket. For the matrix material, a Neo-Hookean type strain energy is selected which depends on the isochoric first invariant of the matrix $\bar{I}_1$, cf. (1)

24 $$\begin{aligned} W^\mathrm {m}= \frac{\mu }{2}(\bar{I}_1^\mathrm {m}-3) \, . \end{aligned}$$

Note that in contrast to the strain energy of collagen, the matrix energy depends on the isochoric part of the invariant. Thus, volume changes do not induce changes in the matrix strain energy. Only the strain energy of collagen yields directions of higher stiffness and can hence via minimization of the potential energy implicitly favor the more compliant directions.

This section is subdivided into three parts. At first, the evolution of the biological species due to damage stimuli and the resulting change of collagen mass are investigated in detail. Afterwards, the chain of healing is presented which is related to damage of tissue followed by the activation of species and resultant tissue growth and remodeling. Hereby, a finite element simulation of a biaxial tension test of a flat tissue sample is conducted. Lastly, a finite element simulation of an indentation test of a tissue sample is presented to shown that the theory can also be applied to more complex, inhomogeneous load cases. The distributed load leads to a distribution of damage in space and thus to a spatially distributed biological response and G&R.

Tables 1,  2 and  3 list the material and simulation parameters for the species study, biaxial tension and indentation test, respectively. The dimension of the flat tissue sample corresponds to measurements in Holzapfel et al. (2005). The volume fraction of non-collageneous matrix and collagen as well as the initial reference mass density are motivated by Humphrey et al. (2014) and Wilson et al. (2012), values of the free energy parameters $\mu$, $k_1$, $k_2$, $\kappa$ are in the range used, e.g., by Gasser and Holzapfel (2002). The damage parameters $m_\mathrm {p}$ and $\bar{\lambda }_\mathrm {p}$ are similar to the one presented by Marino et al. (2019). The homeostatic concentration of MMP and GF are taken from Sáez et al. (2013) and Schaan et al. (2007). The parameters of the species equations are chosen such that the functionality of the model is investigated. In particular, a sensitivity analysis on these latter set of parameters will be conducted in the following Sec. 4.1.

### Biological response at microscale

**Fig. 4:**
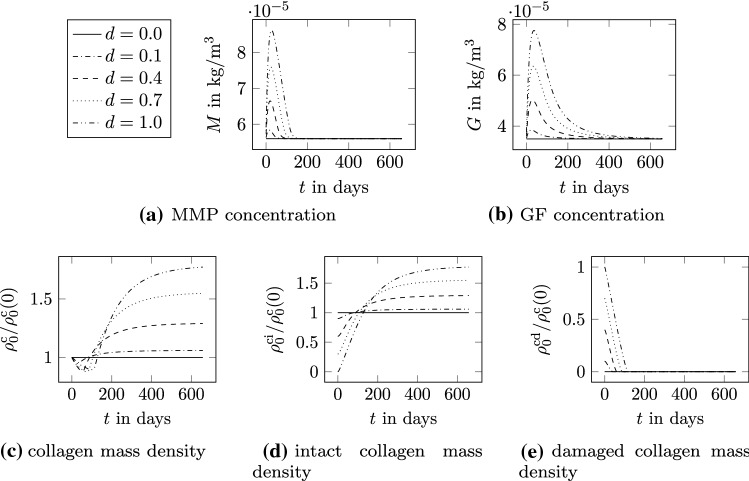
Evolution of *G*, *M*, and $\rho _0^\mathrm {c}$ for different damage values

In this section, the mechanisms at microscale governed by the biological description in (14) - (20) are discussed. The analysis is restricted to a single material point and does not involve any spatial discretization scheme. GF and MMP are released by the presence of damage, which is quantified via the quantity *d*. Although *d* is a variable associated with the mechanical equilibrium problem, it is treated in this first example as a constant predefined quantity. In Fig. 4, the evolution of MMP (Fig. 4a) and GF (Fig. 4b) concentrations as well as the reference density of collagen (Fig. 4c) is depicted for the damage values $d = \left[ 0,0.1,0.4,0.7,1.0 \right]$. The evolution of the collagen density is also shown for the intact (Fig. 4d) and damaged (Fig. 4e) collagen reference density that represent the mass produced and removed due to damage. First of all, it can be observed that the species are only initiated by the presence of damage. For $d=0$, the GF and MMP concentrations remain constant at initial concentrations $G_0$ and $M_0$, respectively. Consequently, also the collagen mass remains constant in that case. As soon as the tissue is damaged, $d>0$, the MMP concentration increases. This leads, on the one hand, to an increase in the GF concentration and, on the other hand, to the removal of damaged tissue. Due to the increase in GF concentration, new tissue is produced. The increase in GF is delayed compared to MMP and hence the removal of damaged tissue is at the beginning higher than the production of new tissue. As a consequence, the tissue mass slightly decreases in the first period of healing. With time, damaged tissue is completely removed. After reaching the maximum concentrations, both MMP and GF concentrations tend back toward the initial concentrations. The proliferation of new collagen continues until the GF concentration equals the initial. At that time, the damaged tissue is already completely removed and hence the intact collagen mass corresponds to the total collagen mass.

The response of the species to damage is more intense for higher damage. The maxima of the MMP and GF concentrations increase, and the species concentrations remain high for a longer period. The more damaged the tissue, the higher is the amount of tissue to be removed and the less intact collagen tissue is present. Looking at the evolution of intact collagen, the production of new mass is higher for a higher amount of damage. However, the production rate is limited due to the exponential term in (20) such that an unlimited increase in mass is prevented. The final value of collagen mass is higher for more damage in the tissue. The rate of removal for damaged collagen increases with the amount of damage. However, the removal rate is limited thanks to the exponential term in (19), leading to the expected outcome that complete removal takes longer when more mass has to be removed. The presented approach can capture both normal and pathological healing. For small damage, e.g., $d=0.1$ in the analysis, collagen turnover yields after approximately 100 days almost the initial collagen mass, reproducing a normal healing condition. For higher damage, collagen mass at the end of healing tends toward higher values with respect to the initial one, determining an abnormal tissue state. It is noteworthy that additional feedback mechanisms, involving for instance the mechanical state, might intervene in the process. These might shift the response from a normal to a pathological one or vice versa. These mechanisms are not considered in the present framework, which can readily be refined when new biological evidence becomes available. However, the possibility of capturing both normal and pathological conditions is a remarkable characteristic showing the potentialities of the present framework.

#### Sensitivity study

**Fig. 5:**
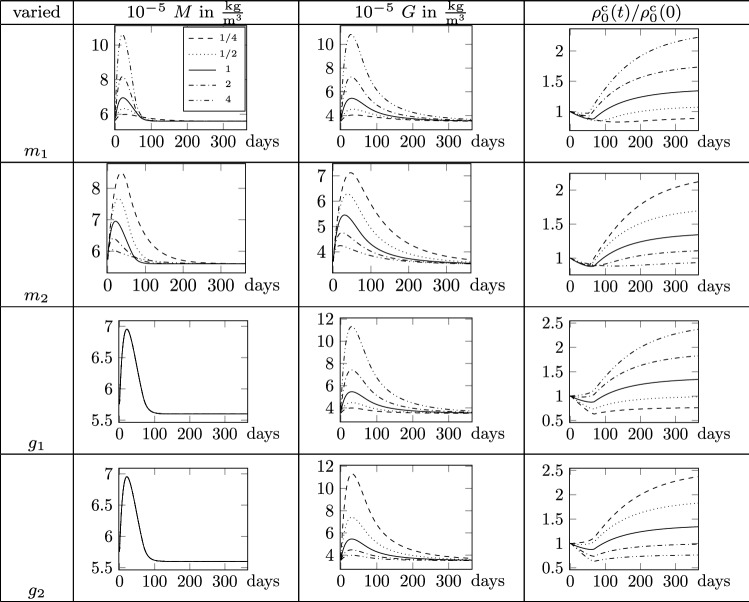
Parameter study of $m_1$, $m_2$, $g_1$, $g_2$ for $d=0.5$. Parameters are varied by factors $1/4, \ldots , 4$ with respect to reference values in Table 1

**Fig. 6:**
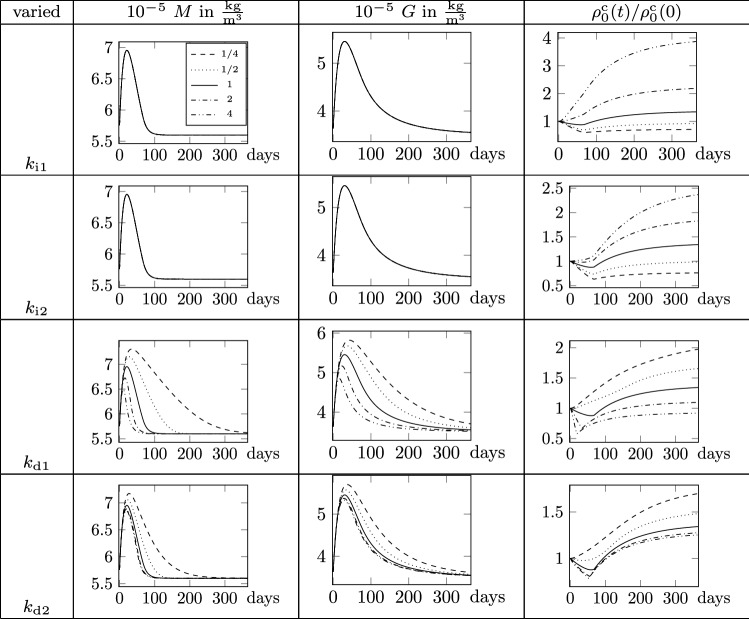
Parameter study of $k_\mathrm {i1}$, $k_\mathrm {i2}$, $k_\mathrm {d1}$, $k_\mathrm {d2}$ for $d=0.5$. Parameters are varied by factors $1/4, \ldots , 4$ with respect to reference values in Table 1

The description of the biological species and collagen mass needs nine parameters. In the following, a sensitivity analysis is performed for eight of those parameters at constant damage $d=0.5$. Omitted is the analysis of the parameter $t_\mathrm {decay}$ which governs the time range within which the GF concentration returns to its homeostatic value, since its effect is clear and undoubtful. It is set constant to $t_\mathrm {decay}=1/2$ year and thus not considered in the parameter study. The analysis is conducted choosing the reference value for each parameter as in Table 1 and varying each parameter at once. Every parameter is decreased by the factors 1/4, 1/2 and increased by 2 and 4, respectively. The results are reported in Figs. 5,  6, showing how variations of the parameters affect the final outcome in terms of normal and pathological healing (including both hypotrophic and hypertrophic collagen production).

Parameters $m_1$ and $m_2$ govern the production and consumption of MMP. The increase in $m_1$ yields an increase in the MMP production and hence a higher maximum MMP concentration. As a consequence, also the GF production increases and more collagen mass is produced. In contrast, increasing the parameter $m_2$ results in a reduction in MMP production rate that leads to less production of GF and thus collagen mass. In addition, the maximum concentration of MMP and GF concentrations is reached earlier in time for higher parameter values. The parameters $g_1$ and $g_2$ govern the production and consumption of GF. Increasing parameter $g_1$ increases the GF concentration and collagen mass, while the MMP concentration is not influenced, cf. Fig. 2. As for the consumption parameter $m_2$ for MMP, the higher the parameter $g_2$ the smaller is the maximum GF concentration and thus collagen mass. Parameters $k_\mathrm {d1}$ and $k_\mathrm {d2}$ govern the removal of damaged collagen. Increasing these parameters yields a faster removal of damaged collagen and since the MMP evolution is a function of the damaged collagen mass, less MMP is produced. Consequently, also the GF concentration decreases and less collagen mass is produced. In addition, an increase in these two parameters shifts the maximum species concentrations toward earlier time points. Furthermore, MMP and thus GF concentrations tend slower back toward the initial value. The variation of $k_\mathrm {d2}$ is in all described effects less sensitive than the variation of $k_\mathrm {d1}$. Lastly, sensitivity of the parameters $k_\mathrm {i1}$ and $k_\mathrm {i2}$, which drive the production of intact collagen mass, is investigated. Both parameters only influence the evolution of the intact collagen mass and yield more collagen mass for higher parameter values.

### Biaxial tension test

**Fig. 7:**
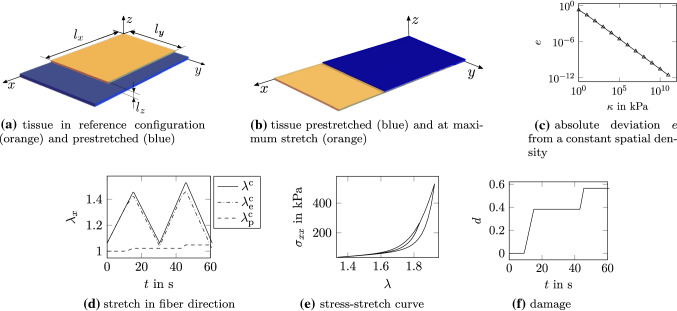
Flat tissue in biaxial tension: damaging phase

**Fig. 8:**
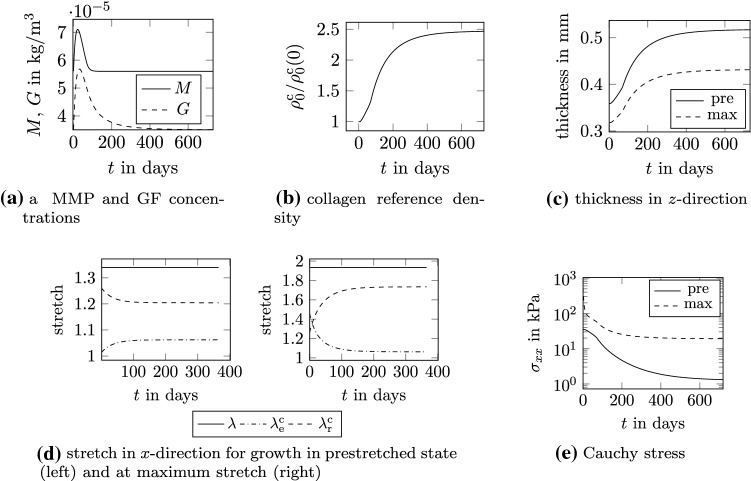
Flat tissue in biaxial tension: response to damage starting from prestretched configuration and at maximum stretch, abbreviations pre: growth in prestretched state, max: growth at maximum stretch

Coupling between the chemo-biological response to damage and the healing-induced growth predicted by the model is investigated by a finite element simulation of a flat tissue sample in a biaxial traction test. The dimensions of the tissue are 12 x 6 x 0.45 mm along the coordinate axes *x*, *y* and *z*. Due to symmetry, only one quarter of the sample is modeled and symmetry boundary conditions are applied at $x=0$, $y=0$ and $z=0$, see Fig. 7a. Due to the homogeneous loading case, the sample is discretized with one H1-P0 element. One set of collagen is considered and aligned along the *x*-direction.

Firstly, prestretch in collagen and the matrix is imposed. Therefore, a stretch of 1.34 in *x*- and 1.25 in *y*-direction is applied and the deformation held constant such that collagen can remodel until the elastic stretch equals a prestretch of 1.062 at the beginning of the damage phase where $t=t_0=0$. These prestretch values correspond to the ones used by Braeu et al. (2017). Secondly, the tissue is damaged during two cycles of biaxial tension in *x*-direction, see Fig. 7b, d. The maximum stretch increases from 1.83 in the first to 1.93 in the second cycle. These values correspond to maximum collagen stretches of 1.45 and 1.53. The threshold stretch in collagen at which damage starts is set to $\bar{\lambda }_\mathrm {p}= 1.3$. Thus, the tissue is already damaged during the first cycle which corresponds to an increase in the damage variable *d*, Fig. 7f, and hence an increase in the plastic stretch in *x*-direction. This yields a reduction in the elastic stretch rate and results in the well-known softening of the stress-stretch response, see Fig. 7e. The significant increase in stress is due to collagen, that carries less load for low stretches but most of the load at high stretches. After the first peak of displacement, the amount of damage and plastic stretch remains constant as long as the maximum stretch is not exceeded.

After the two cycles at $t=t^*=60$ s, the damage in the tissue is constant in time and corresponds to $d^{*} = 0.56$. The damaged and intact collagen mass immediately after the damaging phase are defined as function of the damage variable via (14) and (15).

Due to the presence of damaged collagen, the MMP concentration increases and the species evolution takes its course as described in detail in Sec. 4.1 and shown for the flat tissue in Fig. 8a. Note that the value of the parameter $k_\mathrm {i1}$ is here quite high leading to a mass increase in collagen much larger than it would most likely be observed in reality. This is simply for the sake of illustration. The increase in growth factors induces an increase in collagen mass (Fig. 8b) which in turn translates into a change of the tissue volume.

Two cases are addressed, the healing response occurring either in the prestretched configuration (stretch of 1.34 in *x*- and 1.25 in *y*-direction, Fig. 7a) or in the configuration at maximum stretch (stretch of 1.93 in *x*- and 1.25 in *y*-direction, Fig. 7b). For both cases, the tissue grows in *z*-direction, Fig. 8c, since the displacements in *x*- and *y*-directions are constrained. Although the biological response to damage that drives growth (Fig. 8a and 8b) is the same for the two cases, the samples thicken differently. This difference follows since the sample at maximum stretch needs to thicken less to increase the volume to the same amount as the sample in the prestretched state, and hence to accommodate the same amount of new tissue. Growth in the prestretched state yields an increase in thickness of $44.01 \%$, whereas it increases $35.5 \%$ at maximum stretch. The tissue grown in the configuration at maximum stretch is thinner than the one in the prestretched state. Although these observations are logical, they indicate that the final outcome of growth is strongly influenced by mechanical effects. It illustrates once more the importance of coupling mechanics and biology to appropriately model biological tissues.

Also remodeling evolves differently in the two cases, see Fig. 8d. At the beginning of the healing phase and for healing starting from the prestretched configuration, the elastic collagen stretch is slightly lower than the prestretch value ${\lambda }^\mathrm {c}_\mathrm {pre}$ due to the plastic deformation. Consequently, remodeling leads to an increase in elastic collagen stretch until the elastic collagen stretch in the tissue equals the prestretch. In contrast, at the beginning of the healing phase and for healing at maximum stretch, elastic collagen stretch is 1.53 and thus much higher than the prestretch. As a consequence, tissue remodeling reduces the elastic stretch in collagen and thus also the stress in fiber direction significantly, see Fig. 8e. Although the stretch of the tissue is still the maximum stretch, collagen does not carry much load anymore since highly stretched collagen has been replaced by collagen with a stretch equal to the prestretch. The difference between the total stresses in the two cases is due to the different stress contributions of the isotropic matrix in the two different configurations.

Finally, the effectiveness of enforcing growth through the adopted penalty-based implicit approach is investigated by performing a parametric study on the parameter $\kappa$ in the penalty term $W_\mathrm {L}$ in (21). The results are shown in Fig. 7c, where the error $e(\kappa ,t_\mathrm {end})=|J(\kappa ,t_\mathrm {end})-(\rho _0(t_\mathrm {end}) / \rho _0(0))|$ between the desired and the obtained volume change at the end of the simulation ($t_\mathrm {end}=1$ year) is plotted versus $\kappa$. The choice of $\kappa =10^7 \, \mathrm {J/kg}$ corresponds to an error sufficiently low.

The biaxial test demonstrated the capability of the model to couple damage with the chemo-biological response and growth and remodeling. The evolution of damage due to overstretching was illustrated, and the effect of remodeling during healing was discussed. The next example addresses, on the one hand, the direction of growth and, on the other hand, healing that is not only time dependent but also dependent on the position in space.

### Indentation Test

**Fig. 9:**
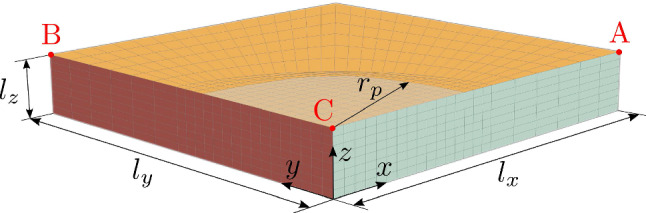
1/4 of a flat tissue sample: a pressure is applied at the top surface in the grey shaded circular area (radius $r_p$)

**Fig. 10:**
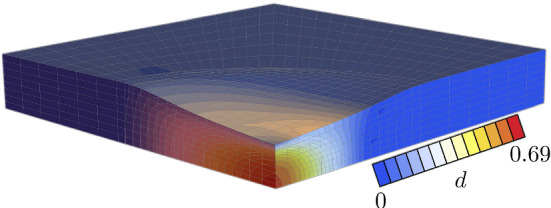
Damage distribution at maximum displacement

**Fig. 11:**
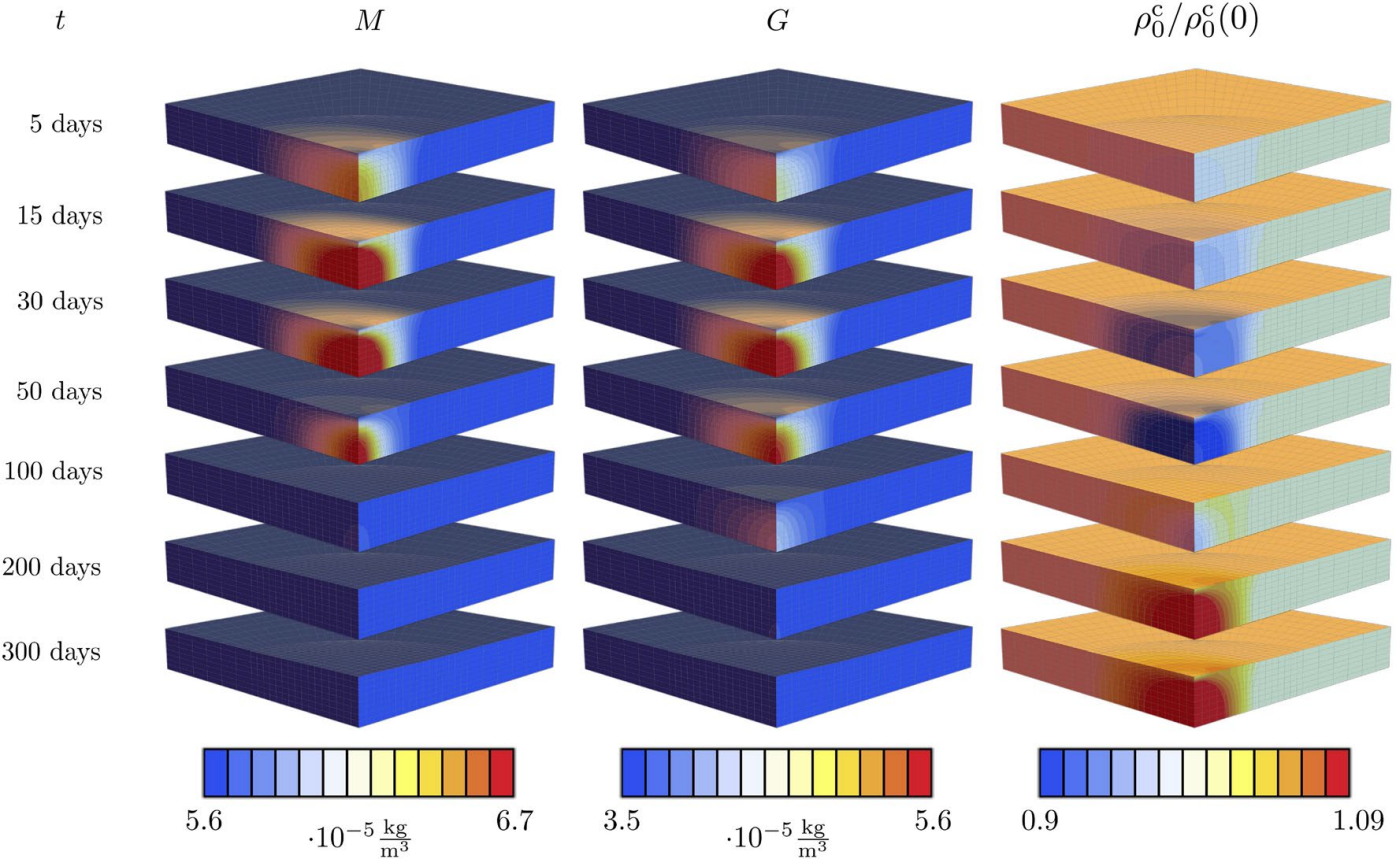
Evolution of MMP, GF and mass throughout one year

**Fig. 12:**
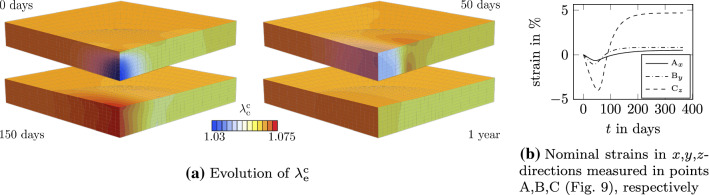
Effect of G&R on $\lambda _\mathrm {e}^\mathrm {c}$ and directions of growth throughout one year

Lastly, the framework is applied to a flat tissue segment that is injured in a circular region at the top surface. Modeled is 1/4 of a plate with symmetry boundary conditions at $x=0$ and $y=0$. Displacement in *z*-direction is prevented at $z=0$. One set of collagen fibers is arranged in the *x*-direction. The geometry is discretized with $22 \times 22 \times 8$ ($x \times y \times z$) H1P0 elements with a finer mesh for the region where the pressure is applied. The geometry and discretization in space are shown in Fig. 9. At $t=t_0=0$, the tissue is already in prestretched state with the procedure introduced in Sec. 4.2 (stretch of 1.34 in *x*- and 1.25 in *y*-direction, corresponding to 1.062 in collagen). In contrast to the biaxial test, starting from $t=t_0$, displacement in *x*- and *y*-direction is permitted allowing growth in all three directions. Therefore, the essential boundary conditions employed to prescribe the desired pre-stretch are replaced by the corresponding traction forces (i.e., natural boundary conditions) at $x=l_x$ and $y=l_y$ (in reference configuration) such that the sample is in equilibrium at $t=t_0$.

For $t_0 \le t \le t^*$, the sample is pressurized at the top surface in a circular region with radius $r_p$ around the point $x=0$, $y=0$, $z=l_z$ with increasing pressure toward the center. In particular, with $r=\sqrt{x^2+y^2}$, a constant pressure in space, $\bar{p}(t)$ is multiplied by the function

25 $$\begin{aligned} f(r) = {\left\{ \begin{array}{ll} 1-\frac{r}{r_p} &{} \text {if} \; r \le 1\,\mathrm {cm} \\ 0 &{} \text {otherwise} \end{array}\right. } \end{aligned}$$

and the pressure $p(r,t)= \bar{p}(t) f(r)$ is applied to induce damage. Maximum damage occurs at $(t_0+t^*)/2$ with a linearly ramped pressure up to $\bar{p}((t_0+t^*)/2)=36$ kPa. The segment is damaged non-uniformly as it can be seen in Fig. 10. The pressure is then completely removed until the end of the damaging phase at $t=t^*=60$ s. The maximum damage of the segment occurs in the center of the specimen, being highly dishomogeneous in all directions. Since fibers are aligned in *x*-direction only, the damage distribution is not the same in *x*- and *y*-direction.

Initiated by the damage stimuli, the species start to evolve at $t=t^*$. In contrast to the previous example in Sec. 4.2, the species evolution is heterogeneous in space, due to the different damage values in space. As discussed in Sec. 4.1, regions with less damage experience a weaker response of the species and thus a smaller increase in mass. To gain a better insight into the heterogeneous evolution of the species and mass, Fig. 11 displays the GF, MMP and collagen mass distributions for discrete time points of the healing process. Due to an increase in the MMP concentration, damaged collagen is removed in the damaged region, whereas the regions without damage remain unchanged. At the same time, the increased GF concentrations yield collagen production. At the beginning, the collagen degradation dominates and hence the total collagen amount of collagen decreases in the damaged region. After approximately 100 days, the MMP concentration is back to the initial concentration and production of collagen dominates such that the initial amount of collagen is exceeded. After 300 days, also the GF concentration approaches the initial concentration. In the whole damage region, collagen mass is higher than initially and peaks where most damage occurred.

The effect of remodeling is addressed in Fig 12a. At the beginning of the healing phase, the elastic collagen stretch is lower in the damaged region due to the plastic deformation. Remodeling yields an increase in the elastic collagen stretch in the damaged region whereby the stretch exceeds the prestretch in some regions (e.g., for $t=50$ days and $t=150$ days). After approximately one year, collagen has remodeled and elastic collagen stretch equals the prestretch.

In this test, growth is permitted in all directions and thus the direction of growth depends on the stiffness of the tissue. The most compliant direction is the *z*-direction in which the tissue can freely grow. The reaction forces due to the prestretch applied at the surface at $y=l_y$ (in reference configuration) yields a stiffer response in *y*-direction and thus growth in this direction is reduced compared to the *z*-direction. Due to the fibers aligned in *x*-direction and the reaction forces applied at $x=l_x$ (in reference configuration), the *x*-direction is the least compliant direction and hence new mass is accommodated least in this direction. The anisotropic growth is illustrated in Fig. 12b, where nominal strains measured at points A, B, C from Fig. 9 are compared to each other. The strains are defined with respect to the configuration at $t=t^*$ to investigate only the deformations due to the healing process. For point A, the strain in *x*-direction, for point B, the strain in *y*-direction and for point C, the strain in *z*-direction is depicted. In all three directions, the strain firstly decreases due to the removal of damaged collagen and increases afterwards due to the addition of newly formed collagen. Among these results, it is mostly interesting to notice that the thickness of the specimen in the damaged area (see Fig. 12b, $C_z$) firstly decreases of $-3.95$ % due to the removal of damaged collagen and then increases of 4.69 % due to the deposition associated with growth and remodeling.

## Limitations

The authors are totally aware of the limitations of the presented model in the perspective of conducting a realistic simulation of soft tissues. However, this paper aims to contribute to the understanding of healing-induced growth and remodeling in soft tissues by addressing challenges arising from the coupling of several mechanisms, both from a theoretical and a computational modeling perspective. The aim of this paper is not to reproduce a specific behavior, and thus all ingredients of the material model are omitted that are not necessary for the presented coupling or for which experimental evidence is still controversial.

Furthermore, only one collagen fiber family is considered although multiple collagen fiber families can be generally observed in several tissues. Due to the convenience of the homogenized constrained mixture framework, other collagen fiber families can be added without much effort since this would readily add further terms in the free energy (10).

Another important limitation is that MMP and GF are here treated as single variables, while several sub-species exist. Moreover, they are considered as local variables, while they might migrate to the side of damage and thus diffusion equations should be added, as, e.g., done by Escuer et al. (2019). In fact, several other species influence the healing of soft biological tissues and might also be key players in the transition from physiological to pathological healing, such as myofibroblasts that are controlled and stimulated by growth factors and contribute significantly to the production of new ECM (Desmoulière et al. 2003). Moreover, an experimental study is clearly necessary to investigate the relationship between interstrand delamination and the initiation of GF, MMP and other species. Furthermore, additional damage mechanisms not related to the collagen molecular level might occur and affect the damage response. These should be incorporated in the modeling framework.

Another limitation, well known for the simulation of biological problems, is the shortage of experimental data. Values of material parameters are chosen within ranges found in the literature, when available. If not available, a parametric study has been presented but the employed values remain uncertain. Furthermore, the behavior of biological tissue differs significantly depending on the age, pathologies and even the location of the tissue in the body. Uncertainties and difficulties of obtaining experimental data clearly remain the biggest issue in the field.

## Conclusion

This paper presents a computational framework to model chemo-mechano-biological mechanisms occurring during the complex healing process in soft biological tissues. The framework uses a multiplicative split of the deformation gradient to account for elastic and inelastic mechanisms at continuum scale. Damage is modeled as a plastic-like mechanism. For the elastoplastic mechanical problem, a set of partial differential equations is solved globally and a set of plastic internal variables is iterated locally at Gauß point level at every time step. Moreover, to determine the concentrations of the molecular species as well as the change of mass, a set of ordinary differential equations is solved locally. These concentrations affect the mass content of constituents, in turn inducing growth observable at the macroscopic level. Remodeling in turn affects the stress-stretch state of the tissue and existent and/or added tissue is replaced by prestretched tissue as long as elastic collagen stretch is different from an assigned prestretch value. The framework is able to capture interactions taking place in the chain of healing. Starting from a homogeneous, pre-stretched state, a measure of interstrand delamination in collagen is taken as damage stimulus that initiates the healing response. Due to damaged tissue, the concentrations of matrix metalloproteinases and growth factors increase, whereby intact collagen is produced and damaged collagen is degraded. Mass turnover of collagen yields, assuming a nearly constant spatial mass density, a change of volume. It was shown that the biological problem bridges from the damage stimulus to the volumetric growth and, depending on the intensity of the damage stimulus as well as the biological response to damage, pathological or physiological healing can be obtained. Furthermore, the sensitivity to parameter changes was addressed. The model is readily extendable for the modeling of further biological species or to incorporate different growth approaches. Furthermore, diffusion of molecular species can (and should) be added.

The outcomes of present paper highlight that more efforts are needed, from both the experimental and the modeling point of view, to gain an insight into the complex healing process of soft tissues, which is highly multifactorial. However, the proposed framework provides a suitable basis for investigating coupled chemo-mechano-biological mechanisms in highly controlled in silico tests which might bring some more pieces to light.
